# A new species and new records of *Molophilus* Curtis, 1833 (Diptera: Limoniidae) from the Western Palaearctic Region

**DOI:** 10.3897/BDJ.3.e5466

**Published:** 2015-08-21

**Authors:** Levente-Péter Kolcsár, Edina Török, Lujza Keresztes

**Affiliations:** ‡ Hungarian Department of Biology and Ecology, Babes-Bolyai University, Cluj-Napoca, Romania; § Romanian Academy Institute of Biology, Bucharest, Romania

**Keywords:** Balkan mountain range, sibling species, distribution

## Abstract

**Background:**

*Molophilus* Curtis, 1833 is the most species-rich Limoniidae genus with a total number of 1006 species and subspecies, from which 97 are recorded in the Western-Palaearctic region so far. However new species are still expected from less investigated regions, like the Balkans or the Eastern Europe.

**New information:**

In the present article, we desrcibe a new limonid crane fly species, *Molophilus balcanicus* Kolcsár **sp. n.** from the Central Balkan area (Bulgaria). This new taxa is closely related to *M. serpentiger* Edwards, 1938 and *M. variispinus* Starý​, 1971 based on the external male genital structures, but differs from its siblings mostly in the structure of the inner and outer gonostylus. Additionally, a number of species are reported for the first time from various European countries, like *M. variispinus* Starý, 1971 and *M. occultus* de Meijere, 1918 from Romania; *M. crassipygus* de Meijere 1918, *M. obsoletus* Lackschewitz, 1940 and *M. medius* de Meijere, 1918 from Greece; *M. flavus* Goetghebuer, 1920 from Andorra; *M. cinereifrons* de Meijere, 1920 from Bulgaria and *M. corniger* Meijere, 1920 from Spain.

## Introduction

*Molophilus* Curtis, 1833 is the most species-rich genus in the Western-Palaearctic region, belonging to Limoniidae (Diptera). The species can be easily identified based on male robust hypopigium. The females are unknown or less studied. So far, there are more than 1000 species and subspecies are described worldwide with an Australasian-Oceanian distribution center (Oosterbroek 2015). At the present 97 species and subspecies are known to occur in the Western-Palaearctic region (Oosterbroek 2015, Starý 2011). In the present study we report 8 new faunistic data from different countries in Europe, and we describe a new species which is closely related to *M. serpentiger* Edwards, 1938 and *M. variispinus* Starý, 1971. Both of the two closely related species, *M. serpentiger* and *M. variispinus* share a unique feature within the genus *Molophilus*, the presence of an S-shaped outer gonostylus (Starý 1971b, Starý 1971a), which is highly similar in *M. variispinus* and *M. serpentiger* (mostly seen in ventral view) (Kramer 2013). In the frame of the current study we present the habitus, the male hypogium (ventral and lateral view) and the aedeagal complex, both in the case of *M. serpentiger*, *M. variispinus* and *M. balcanicus* Kolcsár **sp. n**.

## Materials and methods

The material was collected by entomological net and UV light trap in Andorra, Bulgaria, Greece, Romania and Spain, beetwen 2010 and 2013. All the material listed here, are stored in 96% ethanol and deposited in the Diptera Collection of the Faculty of Biology and Geology, Cluj-Napoca, Romania. The holotype of the new species is deposited in the Museum of Zoology of the Babeș-Bolyai University (MZBBU), Cluj-Napoca, Romania. The specimens were examined with an Olympus SZ50 dissection microscope. Photos were taken using an Canon EOS 650D digital camera, attached to an Olympus SZ60 stereomicroscope, with a LM Digital SLR Adapter (Micro Tech Lab, Austria). Layer photos were finally combined with the software Combine ZP (Hadley 2011).

## Taxon treatments

### Molophilus (Molophilus) balcanicus

Kolcsár sp. n.

urn:lsid:zoobank.org:act:31EEC292-24B6-40B4-93C9-9669C047AE2E

#### Materials

- **Type status:** Holotype. **Occurrence:** catalogNumber: TI100 - LCMMB-BG-01; recordedBy: L. Keresztes, E. Török, L.-P. Kolcsár; individualCount: 1; sex: male; **Taxon:** genus: Molophilus; subgenus: Molophilus; specificEpithet: balcanicus; scientificNameAuthorship: Kolcsár; **Location:** country: Bulgaria; stateProvince: Montana; county: Berkovitza; municipality: Barzia; locality: Petrohan Pass; verbatimElevation: 1100-1200 m; verbatimCoordinateSystem: decimal degrees; verbatimSRS: WGS84; decimalLatitude: 43.134061; decimalLongitude: 23.149889; **Event:** samplingProtocol: butterfly net; eventDate: 06/10/2012; habitat: small brook in beech forest; **Record Level:** institutionCode: Museum of Zoology, Babeș-Bolyai University (MZBBU)

#### Description

**Head.** Vertex black, with short black setae. Rostrum light brown with a few short dark setae. Palpus 4-segmented, brown. Antennae 16-segmented, yellowish, only the scape is dark brown. Pedicellus globular 1.5-1.8x wider than the flagellum segments. Flagellomeres are cylindrical to fusiform.

**Thorax.** Yellowish orange (Fig. 1a​). Frontal parts of the thorax are darker than the back parts, with an evenly transition from yellow to dark orange. The neck (cervix) has its lateral parts black (cervical sclerites). Prescutum and scutum orange, scutellum yellow, pleural part yellow - orange. The coxae and the trochanters are yellowish orange, with long pale setae. The femur, tibia and tarsomeres are absent in the examined material. Wings yellow, wing venation light brown, covered by a densely light brown macrotrichia, partly worn out in specimen examined by us. Haltereres are yellow.

**Abdomen.** Dark orange - light brown, tergites mainly dark, anterior sternites lighter than caudal sternites. Both sternites and tergites are covered with long pale setae. Pleural membrane yellow. **Hypopigium** generally yellow (Fig. 1b, c). 9th tergite covered by long pale setae. The dorsal portion of the gonocoxite is short, rounded in lateral view, the caudal margine is straight, with a darkened inner wedge like projection at the ventral edge, which is striking dark pigmented at the end (ventral (tergal) view - Fig. 1b). Ventral lobe of gonocoxite shorter than dorsal portion and it is rounded at the tip. All parts of gonocoxit are covered by long yellowish setae. Both gonostyli are darkly pigmented. The inner gonostylus has its proximal part very thick, which ventrally narrows and is slightly curved. The outer gonostylus is thick, S-shaped inwards and it is ending in a hook like structure ventrally. The proximal part is stouter and lighter than distally, which widens before the hook like end (ventral view - Fig. 1b). Aedeagus is long, the end reaches the gonocoxite apex. The proximal part is thick which narrows to the distal end. In half of the length of the aedeagus is curved ventrally, after that it turns dorsally (Fig. 1d​​).

**Female**: unknown.

**Larva**: unknown.

#### Diagnosis

Small species with yellowish orange general colour, body lenght is 4-4.5 mm and wing length 5 mm (Fig. 1a​​). It is very close to *Molophilus serpentiger* and *M. variispinus*. Inner gonostylus thick, narrows to end. Outer gonostylus thick S-haped having a hook like end.

#### Etymology

*Balcanicus* (latin) = referring to the Balkan area, from where the species were collected. The name is to be deemed to be a latinized adjective in nominative singular.

#### Distribution

Bulgaria (Balkan mountain range).

#### Ecology

Only one specimen was collected in a beech forest, close to a small brook at 1100-1200 m. The valley of the brook was dominated by medium to large sized rocks, along with thick layer of accumulated leaf litter.

#### Taxon discussion

The new species is very close to *Molophilus serpentiger* and *M. variispinus*, but differs from both sibling species by the thick outer and inner gonostylus. Both of the two already known sibling species have thin outer gonostylus without hook like end (Figs 1c, 2c, 3c​​). *M. balcanicus* **sp. n**. is more close related to *M. variispinus*, than to *M. *serpentiger**, on the base of the shape of the aedeagal complex (Figs 1d, 2d, 3d​).

### Molophilus (Molophilus) variispinus

Starý, 1971

#### Materials

- **Type status:** Other material. **Occurrence:** catalogNumber: LCMMVa-RO-01; recordedBy: L.-P. Kolcsár; individualCount: 1; sex: male; **Taxon:** genus: Molophilus; subgenus: Molophilus; specificEpithet: variispinus; scientificNameAuthorship: Starý, 1971; **Location:** country: Romania; stateProvince: Harghita; municipality: Izvoare; locality: Izvoare Valley; verbatimElevation: 1415 m; verbatimCoordinateSystem: decimal degrees; verbatimSRS: WGS84; decimalLatitude: 46.454143; decimalLongitude: 25.56545; **Event:** samplingProtocol: butterfly net; eventDate: 07/10/2013; habitat: brook in spruce (*Picea*) forest
- **Type status:** Other material. **Occurrence:** catalogNumber: LCMMVa-RO-02; recordedBy: L.-P. Kolcsár; individualCount: 2; sex: male; **Taxon:** genus: Molophilus; subgenus: Molophilus; specificEpithet: variispinus; scientificNameAuthorship: Stary, 1971; **Location:** country: Romania; stateProvince: Harghita; municipality: Liban; locality: Lacul Dracului bog; verbatimElevation: 1175 m; verbatimCoordinateSystem: decimal degrees; verbatimSRS: WGS84; decimalLatitude: 46.548137; decimalLongitude: 25.587024; **Event:** samplingProtocol: butterfly net; eventDate: 06/27/2013; habitat: bog in spruce (*Picea*) forest

#### Notes

First record to Romania. The habitus, the hypopigium and the aedeagal complex of the male are illustrated in ​​Fig. 2.

### Molophilus (Molophilus) serpentiger

Edwards, 1938

#### Materials

- **Type status:** Other material. **Occurrence:** catalogNumber: LCMMSe-RO-01; recordedBy: L. Keresztes, L.-P. Kolcsár; individualCount: 1; sex: male; **Taxon:** genus: Molophilus; subgenus: Molophilus; specificEpithet: serpentiger; scientificNameAuthorship: Edwards, 1938; **Location:** country: Romania; stateProvince: Maramures; municipality: Borșa; locality: Vișeu River; verbatimElevation: 980 m; verbatimCoordinateSystem: decimal degrees; verbatimSRS: WGS84; decimalLatitude: 47.62293; decimalLongitude: 24.809762; **Event:** samplingProtocol: butterfly net; eventDate: 05/17/2013; habitat: small brook in spruce (*Picea*) forest

#### Notes

The habitus, the hypopigium and the aedeagal complex of the male are illustrated in Fig. 3.

### Molophilus (Molophilus) cinereifrons

de Meijere, 1920

#### Materials

- **Type status:** Other material. **Occurrence:** catalogNumber: LCMMCi-BG-01; recordedBy: E. Török, L. Keresztes, L.-P. Kolcsár; individualCount: 1; sex: male; **Taxon:** genus: Molophilus; subgenus: Molophilus; specificEpithet: cinereifrons; scientificNameAuthorship: de Meijere, 1920; **Location:** country: Bulgaria; stateProvince: Troyan; municipality: Beli Osam; locality: Troyan Pass; verbatimElevation: 1468 m; verbatimCoordinateSystem: decimal degrees; verbatimSRS: WGS84; decimalLatitude: 42.781119; decimalLongitude: 24.613081; **Event:** samplingProtocol: butterfly net; eventDate: 06/12/2012; habitat: small spring

#### Notes

First record to Bulgaria.

### Molophilus (Molophilus) corniger

de Meijere, 1920

#### Materials

- **Type status:** Other material. **Occurrence:** catalogNumber: LCMMCo-ES-01; recordedBy: M. Bálint; individualCount: 2; sex: male; **Taxon:** genus: Molophilus; subgenus: Molophilus; specificEpithet: corniger; scientificNameAuthorship: de Meijere, 1920; **Location:** country: Spain; stateProvince: La Rioja; municipality: Ezcaray; locality: Valdezcaray ski area; verbatimElevation: 1620 m; verbatimCoordinateSystem: decimal degrees; verbatimSRS: WGS84; decimalLatitude: 42.255772; decimalLongitude: -2.97818; **Event:** samplingProtocol: butterfly net; eventDate: 07/24/2012; habitat: small spring

#### Notes

First record to Spain.

### Molophilus (Molophilus) crassipygus

de Meijere, 1918

#### Materials

- **Type status:** Other material. **Occurrence:** catalogNumber: LCMMCr-GR-01; recordedBy: L. Rákosy; individualCount: 1; sex: male; **Taxon:** genus: Molophilus; subgenus: Molophilus; specificEpithet: ​crassipygus; scientificNameAuthorship: de Meijere, 1918; **Location:** country: Greece; municipality: Ioanina; locality: Tymfi Mts.; verbatimElevation: 1750 m; verbatimCoordinateSystem: decimal degrees; verbatimSRS: WGS84; decimalLatitude: 39.942732; decimalLongitude: 20.838062; **Event:** samplingProtocol: UV light trap; eventDate: 08/05/2012

#### Notes

First record to Greece.

### Molophilus (Molophilus) flavus

Goetghebuer, 1920

#### Materials

- **Type status:** Other material. **Occurrence:** catalogNumber: LCMMF-AND-01; recordedBy: M. Bálint; individualCount: 1; sex: male; **Taxon:** genus: Molophilus; subgenus: Molophilus; specificEpithet: flavus; scientificNameAuthorship: Goetghebuer, 1920; **Location:** country: Andorra; stateProvince: La Massana; municipality: Pal; verbatimElevation: 1910 m; verbatimCoordinateSystem: decimal degrees; verbatimSRS: WGS84; decimalLatitude: 42.533972; decimalLongitude: 1.465611; **Event:** samplingProtocol: butterfly net; eventDate: 07/17/2012; habitat: small brook

#### Notes

First record to Andorra.

### Molophilus (Molophilus) medius

de Meijere, 1918

#### Materials

- **Type status:** Other material. **Occurrence:** catalogNumber: LCMMM-BG-01; recordedBy: L. Rákosy; individualCount: 16; sex: male; **Taxon:** genus: Molophilus; subgenus: Molophilus; specificEpithet: medius; scientificNameAuthorship: de Meijere, 1918; **Location:** country: Greece; municipality: Ioanina; locality: Tymfi Mts.; verbatimElevation: 1750 m; verbatimCoordinateSystem: decimal degrees; verbatimSRS: WGS84; decimalLatitude: 39.942732; decimalLongitude: 20.838062; **Event:** samplingProtocol: UV light trap; eventDate: 08/05/2012

#### Notes

First record to Greece.

### Molophilus (Molophilus) obsoletus

Lackschewitz, 1940

#### Materials

- **Type status:** Other material. **Occurrence:** catalogNumber: LCMMOb-GR-01; recordedBy: L. Rákosy; individualCount: 3; sex: male; **Taxon:** genus: Molophilus; subgenus: Molophilus; specificEpithet: obsoletus; scientificNameAuthorship: Lackschewitz, 1940; **Location:** country: Greece; municipality: Ioanina; locality: Tymfi Mts.; verbatimElevation: 1750 m; verbatimCoordinateSystem: decimal degrees; verbatimSRS: WGS84; decimalLatitude: 39.942732; decimalLongitude: 20.838062; **Event:** samplingProtocol: UV light trap; eventDate: 08/05/2012

#### Notes

First record to Greece.

### Molophilus (Molophilus) occultus

de Meijere, 1918

#### Materials

- **Type status:** Other material. **Occurrence:** catalogNumber: LCMMO-RO-01; recordedBy: L. Keresztes; individualCount: 1; sex: male; **Taxon:** genus: Molophilus; subgenus: Molophilus; specificEpithet: occultus; scientificNameAuthorship: de Meijere, 1918; **Location:** country: Romania; stateProvince: Maramures; municipality: Borșa; locality: Cascada Cailor; verbatimElevation: 1350 m; verbatimCoordinateSystem: decimal degrees; verbatimSRS: WGS84; decimalLatitude: 47.586801; decimalLongitude: 24.668921; **Event:** samplingProtocol: butterfly net; eventDate: 06/26/2010; habitat: spruce ​(*Picea*) forest

#### Notes

First record to Romania.

## Supplementary Material

XML Treatment for Molophilus (Molophilus) balcanicus

XML Treatment for Molophilus (Molophilus) variispinus

XML Treatment for Molophilus (Molophilus) serpentiger

XML Treatment for Molophilus (Molophilus) cinereifrons

XML Treatment for Molophilus (Molophilus) corniger

XML Treatment for Molophilus (Molophilus) crassipygus

XML Treatment for Molophilus (Molophilus) flavus

XML Treatment for Molophilus (Molophilus) medius

XML Treatment for Molophilus (Molophilus) obsoletus

XML Treatment for Molophilus (Molophilus) occultus

## Figures and Tables

**Figure 1a.**
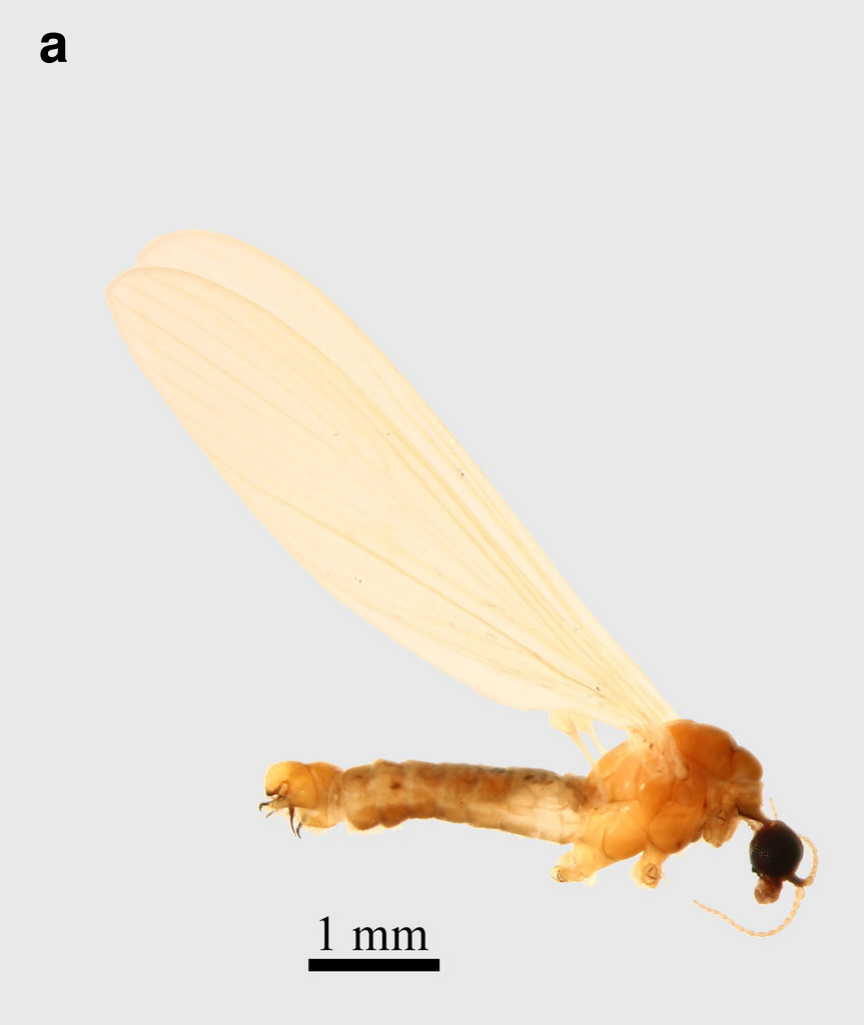
male habitus

**Figure 1b.**
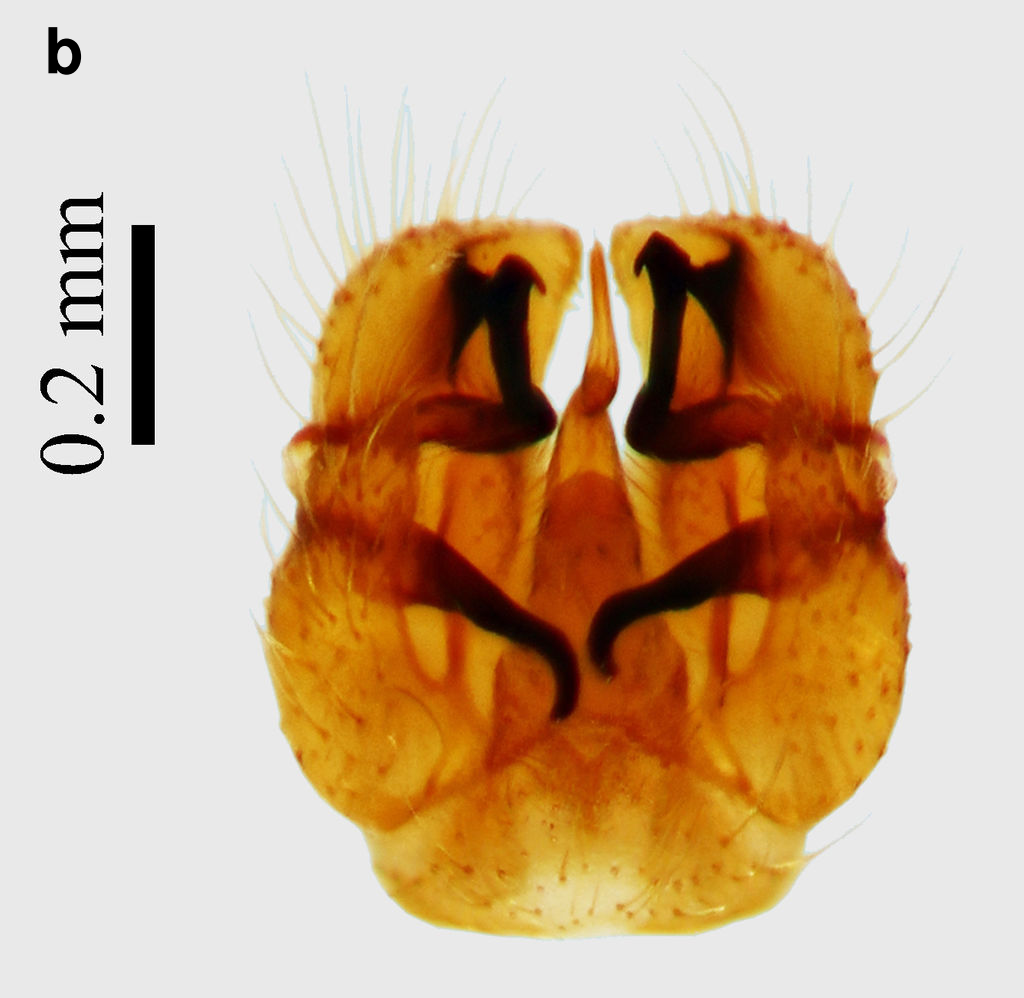
male hypopygium, ventral (tergal) view

**Figure 1c.**
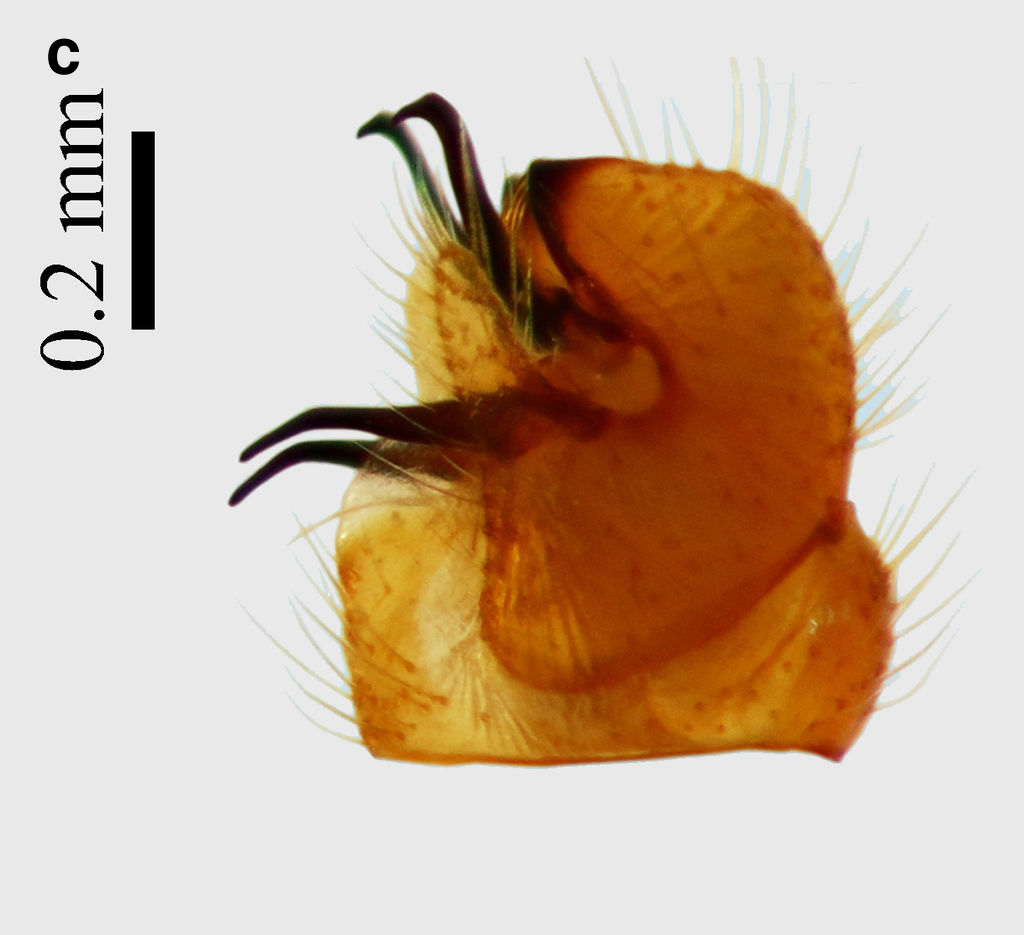
male hypopygium, lateral view

**Figure 1d.**
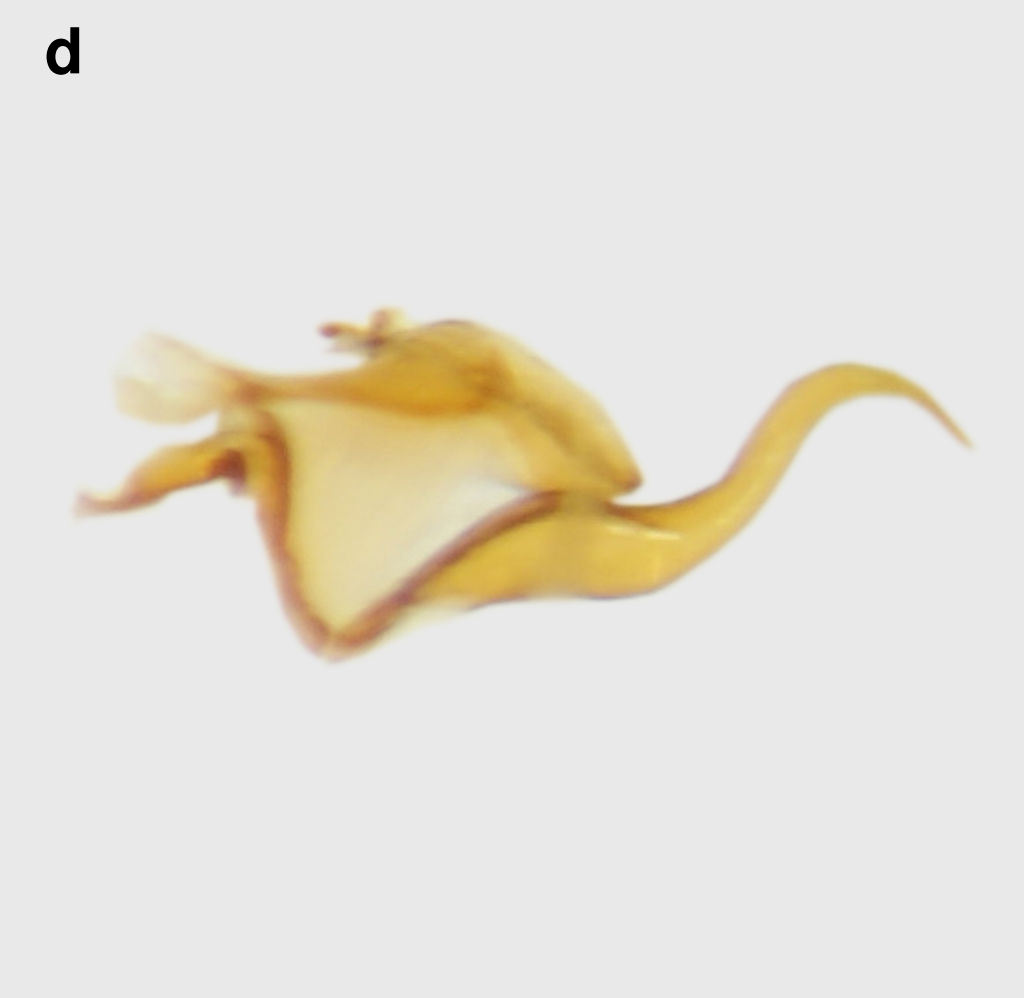
aedeagal complex, lateral view

**Figure 2a.**
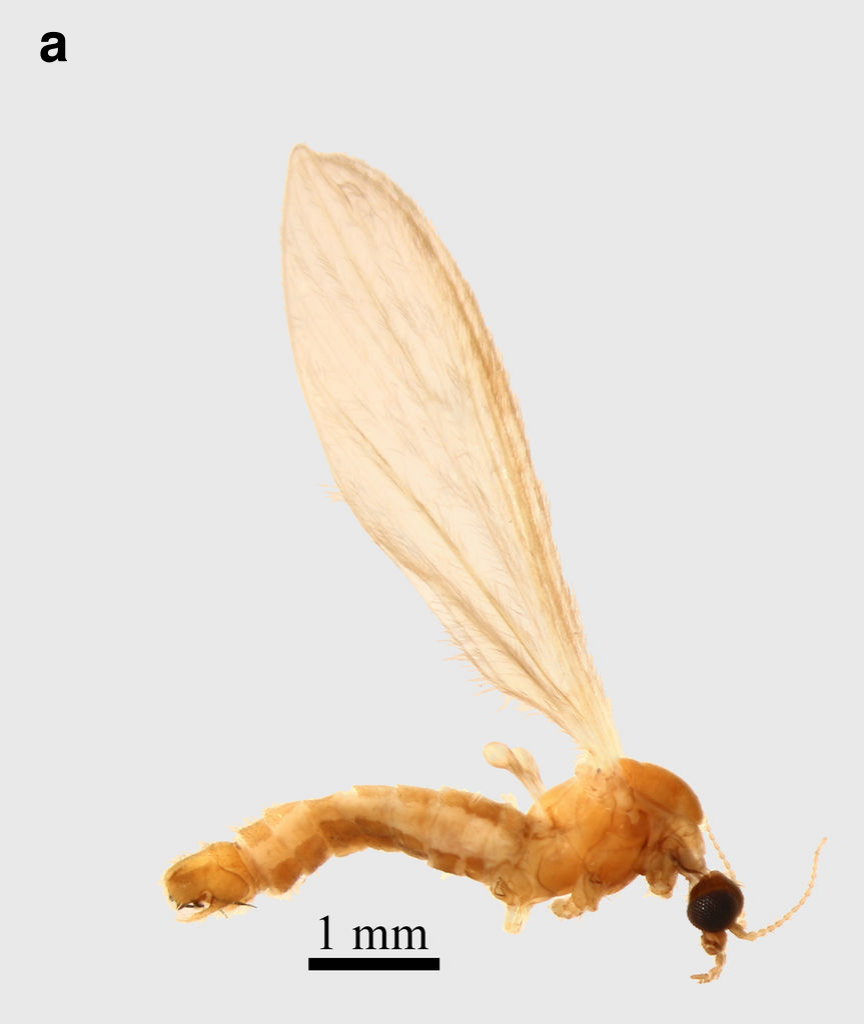
male habitus

**Figure 2b.**
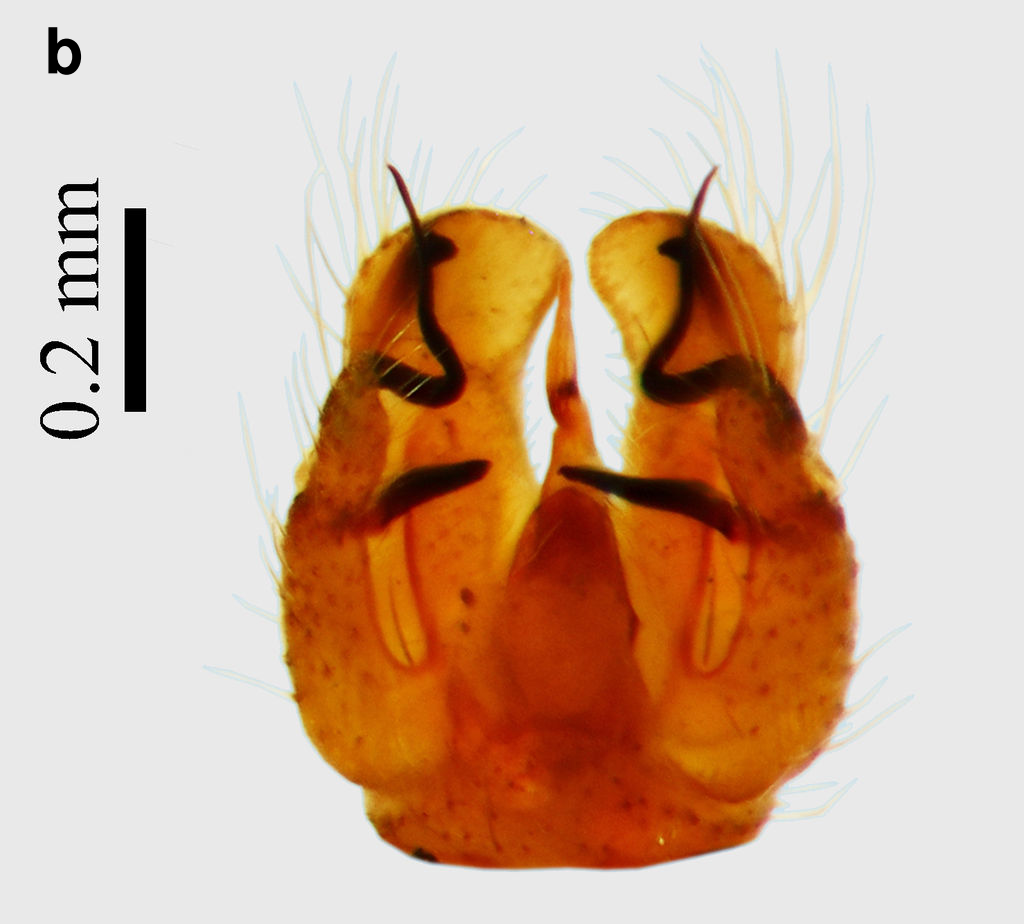
male hypopygium, ventral (tergal) view

**Figure 2c.**
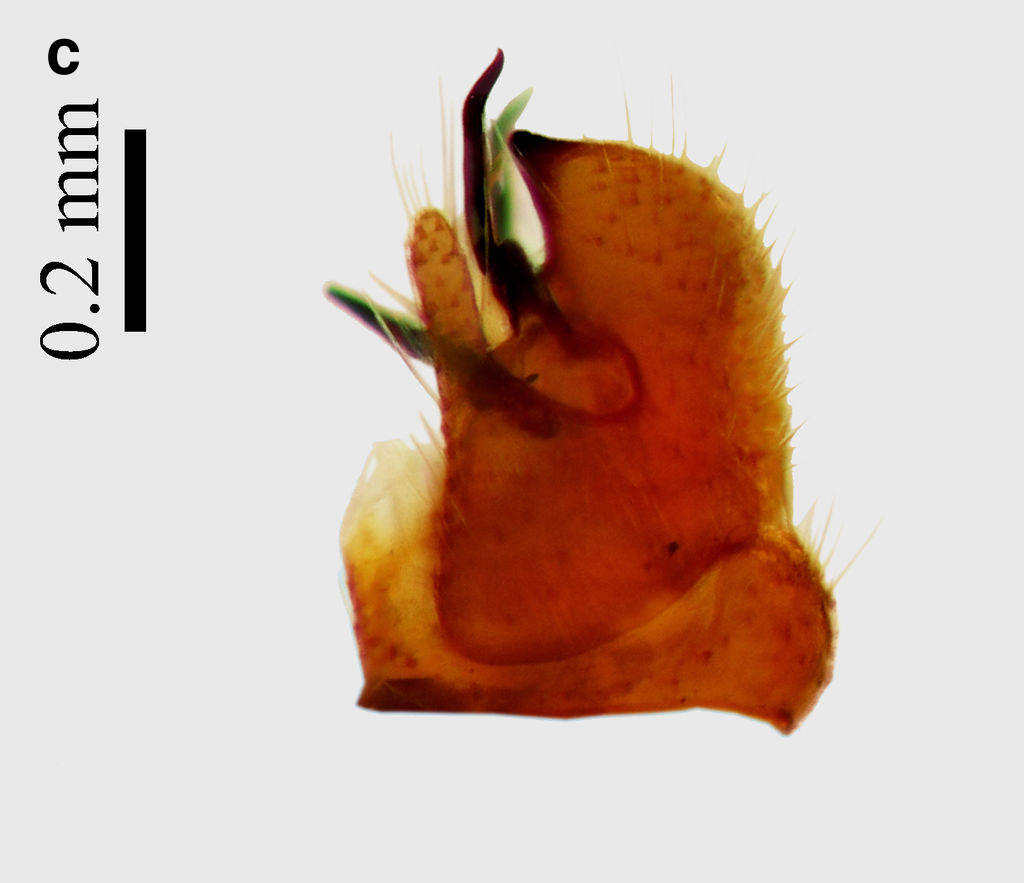
male hypopygium, ventral (tergal) view

**Figure 2d.**
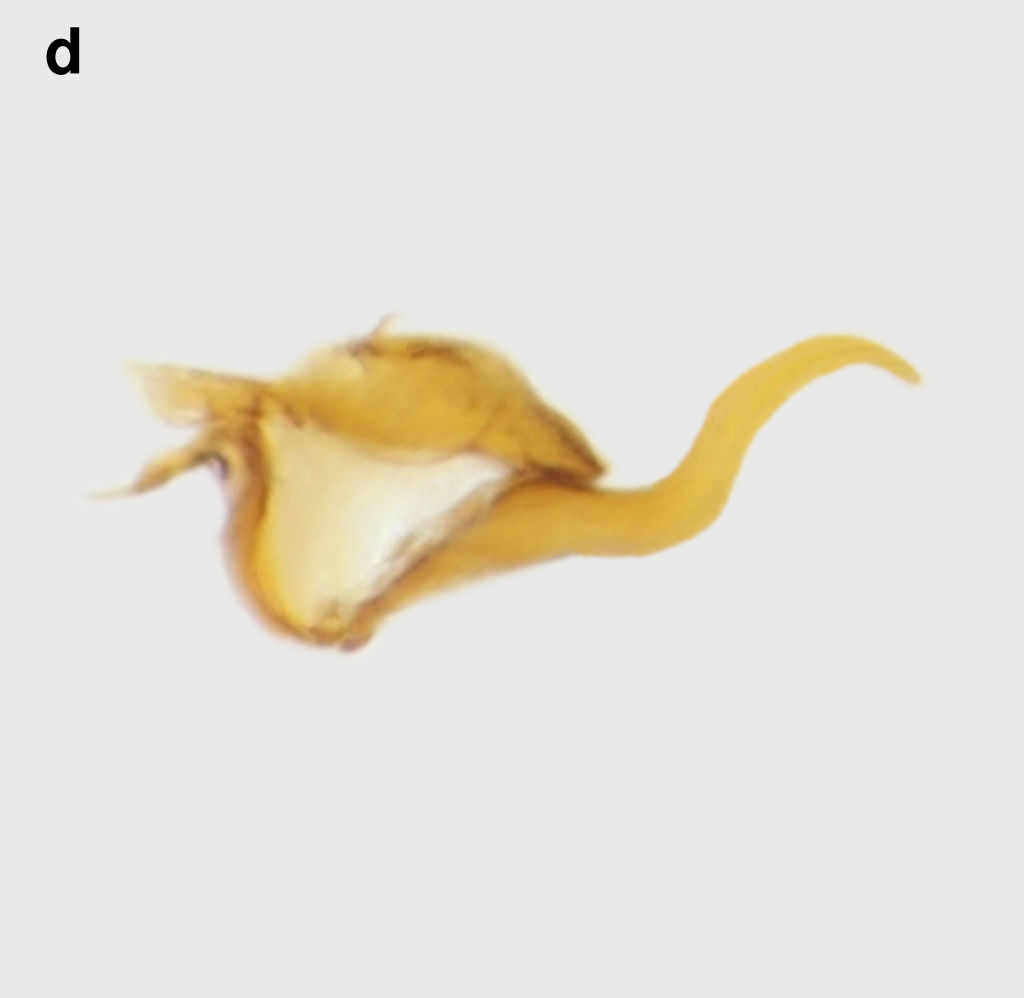
aedeagal complex, lateral view

**Figure 3a.**
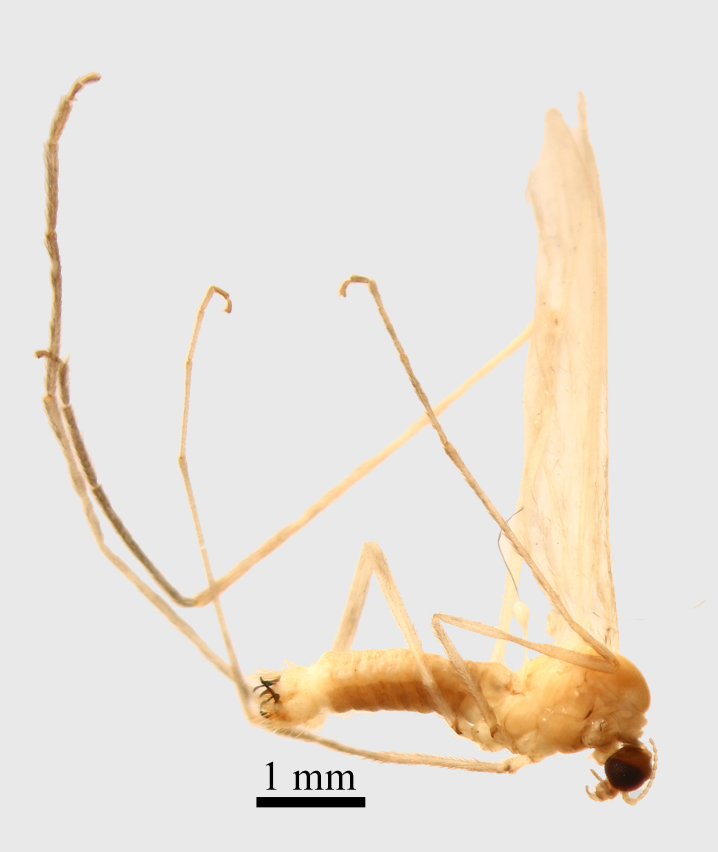
male habitus

**Figure 3b.**
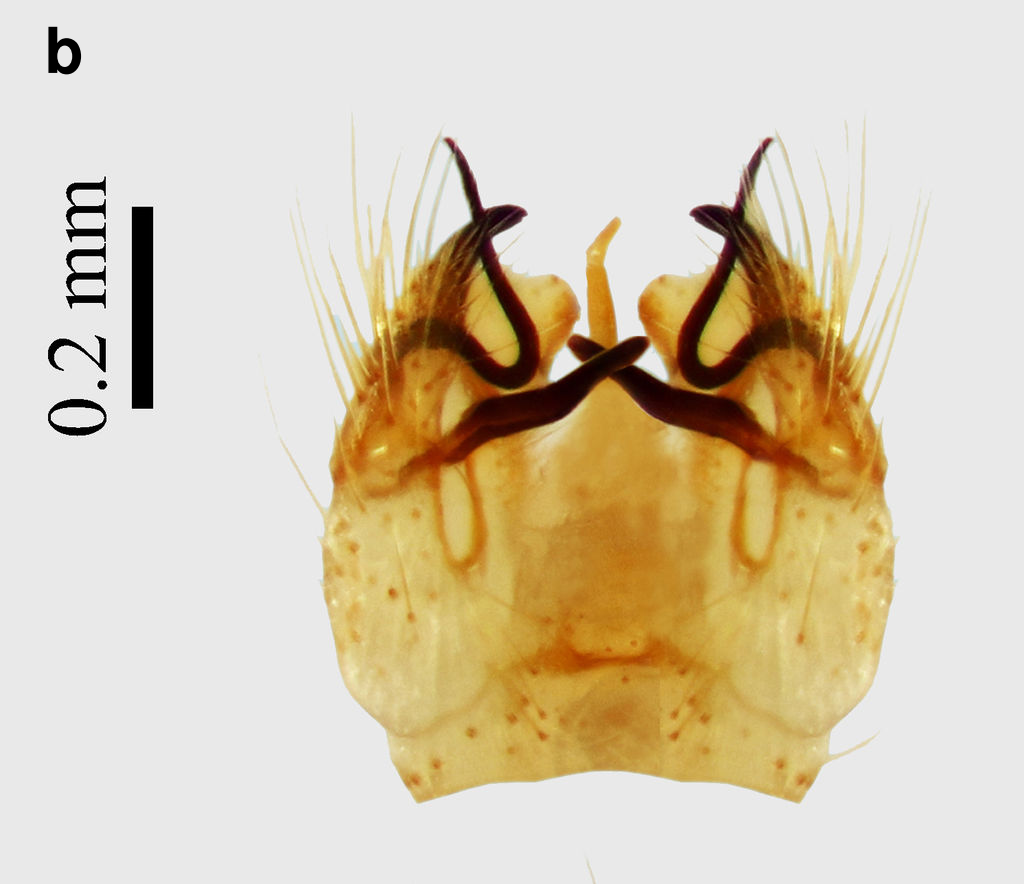
male hypopygium, ventral (tergal) view

**Figure 3c.**
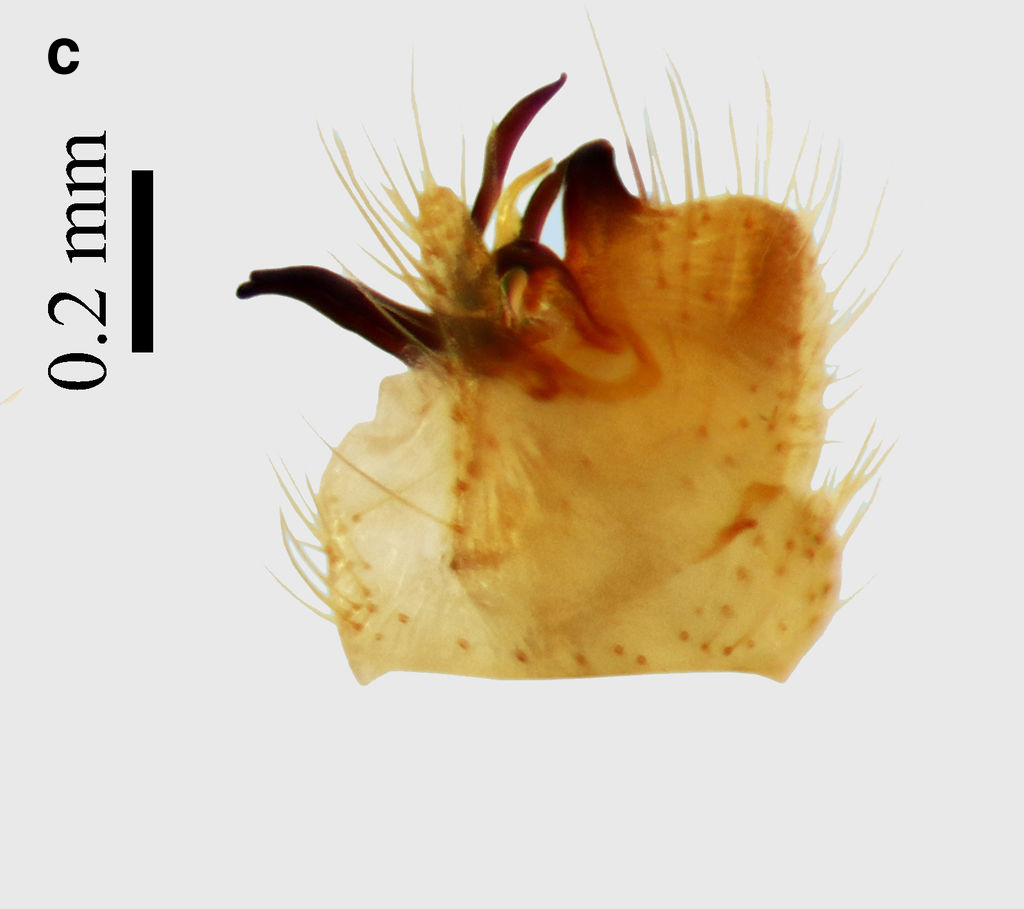
male hypopygium, lateral view

**Figure 3d.**
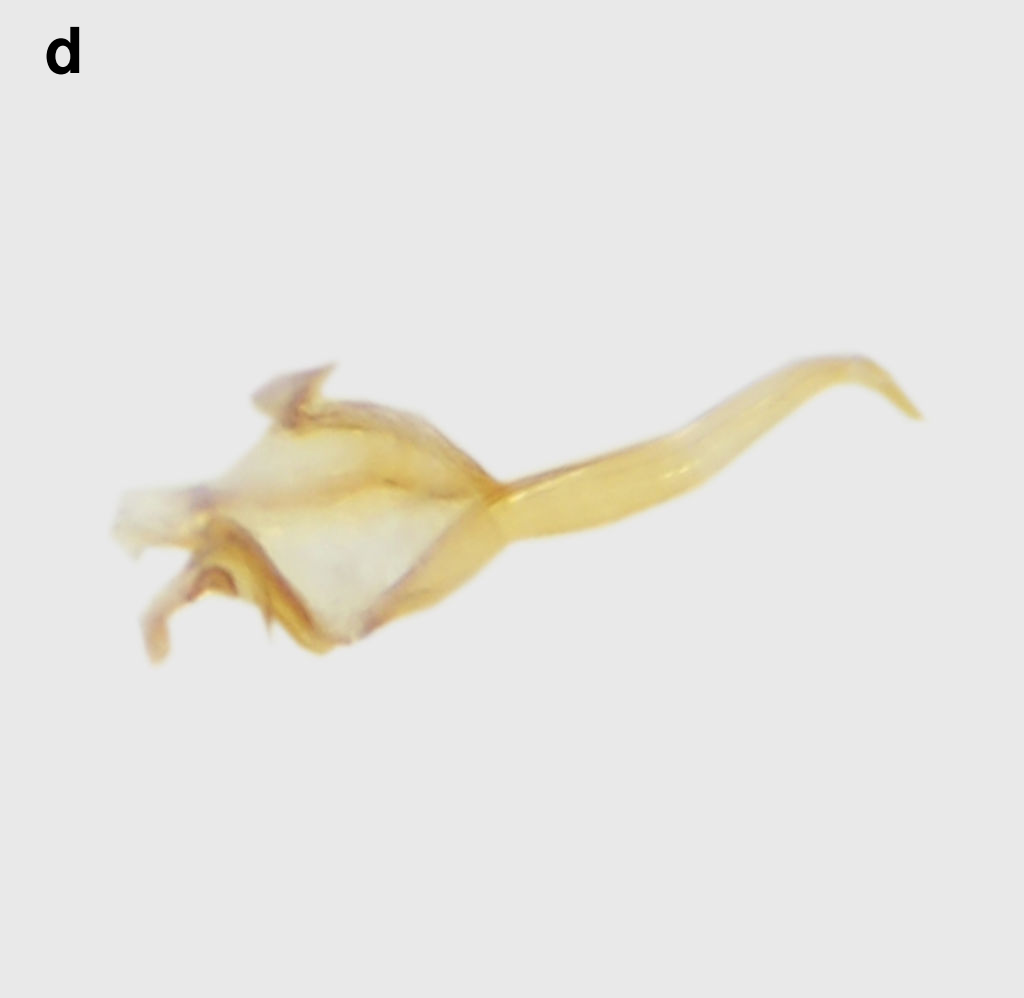
aedeagal complex, lateral view
